# Identifying circRNA-associated-ceRNA networks in the hippocampus of Aβ1-42-induced Alzheimer's disease-like rats using microarray analysis

**DOI:** 10.18632/aging.101427

**Published:** 2018-04-27

**Authors:** Zhe Wang, Panpan Xu, Biyue Chen, Zheyu Zhang, Chunhu Zhang, Qiong Zhan, Siqi Huang, Zi-an Xia, Weijun Peng

**Affiliations:** 1 Department of Integrated Traditional Chinese and Western Medicine, The Second Xiangya Hospital, Central South University, Changsha, Hunan 410011, China; 2 Department of Integrated Traditional Chinese and Western Medicine, Xiangya Hospital, Central South University, Changsha, Hunan 410008, China; 3 Department of Neurology, The Second Xiangya Hospital, Central South University, Changsha, Hunan 410011, China

**Keywords:** Alzheimer’s disease, hippocampus, Aβ1-42, ceRNA networks, microarray expression profile

## Abstract

Alzheimer’s disease (AD) is the most common form of dementia worldwide. Accumulating evidence indicates that non-coding RNAs are strongly implicated in AD-associated pathophysiology. However, the role of these ncRNAs remains largely unknown. In the present study, we used microarray analysis technology to characterize the expression patterns of circular RNAs (circRNAs), microRNAs (miRNAs), and mRNAs in hippocampal tissue from Aβ_1-42_-induced AD model rats, to integrate interaction data and thus provide novel insights into the mechanisms underlying AD. A total of 555 circRNAs, 183 miRNAs and 319 mRNAs were identified to be significantly dysregulated (fold-change ≥ 2.0 and *p*-value < 0.05) in the hippocampus of AD rats. Quantitative real-time polymerase chain reaction (qRT-PCR) was then used to validate the expression of randomly-selected circRNAs, miRNAs and mRNAs. Next, GO and KEGG pathway analyses were performed to further investigate ncRNAs biological functions and potential mechanisms. In addition, we constructed circRNA-miRNA and competitive endogenous RNA (ceRNA) regulatory networks to determine functional interactions between ncRNAs and mRNAs. Our results suggest the involvement of different ncRNA expression patterns in the pathogenesis of AD. Our findings provide a novel perspective for further research into AD pathogenesis and might facilitate the development of novel therapeutics targeting ncRNAs.

## Introduction

Alzheimer’s disease (AD), the most common cause of dementia worldwide, is becoming more prevalent due to the aging population, and represents one of the grand challenges to health care systems [1]. Although substantial progress has been made in the identification of disease-related molecular and cellular processes over the last decade, the molecular mechanisms that underlie the pathogenesis of AD remain largely unknown [2], and none of the pharmacological treatments presently available for AD are able to stop or slow down the progression of this disease [3].Therefore, further investigation of the underlying disease mechanisms are urgently required in order to better understand AD and to facilitate the development of effective therapeutic strategies.

Currently, accumulating evidence indicates that noncoding RNAs (ncRNAs), especially microRNAs (miRNAs), long non-coding RNAs (lncRNAs), and circular RNAs (circRNAs), are strongly implicated in AD-associated pathophysiology, including amyloid-β plaques and neurofibrillary tangles, synaptic loss and neuronal death [4,5]. In a previous study, we investigated the hippocampal expression patterns of dysregulated lncRNAs in a rat model of AD using microarray analysis and demonstrated that lncRNAs contributed to the pathogenesis of AD [6]. To further investigate the regulatory role of ncRNAs in AD, we focused upon circRNAs, a class of ncRNAs that are highly expressed in the mammalian brain [7,8], and can regulate transcriptional and post-transcriptional gene expression [9]. Unlike linear RNA, circRNA is formed with covalently closed continuous loops without 5’-3’ polarity and a poly(A) tail, and might function as microRNA sponges to modulate the expression of parental genes through the competing endogenous RNA (ceRNA) network [10]. Recent studies have provided evidence that the circRNA-associated ceRNA network may play a crucial role in many disease processes, including AD [11]. For example, Lukiw et al. [12,13] demonstrated that circRNA-7(ciRS-7) acted as a natural miRNA sponge for miRNA-7 and regulated the expression of ubiquitin-conjugating enzyme E2A (UBE2A) and the epidermal growth factor receptor (EGFR) in sporadic AD hippocampal brain. Zhang et al. [14] characterized circRNA-associated-ceRNA networks in the cerebral cortex of senescence-accelerated mouse prone 8(SAMP8). Additionally, Zhong et al. [15] described the expression of circRNAs in different ages of SAMP8 mice. However, the potential role of circRNAs in the pathogenesis of AD is still in its infancy and has yet to be characterized, particularly the role of circRNA-associated-ceRNA networks in the hippocampus of AD.

In the present study, we used a microarray analysis approach to identify differentially-expressed profiles of circRNAs, miRNAs and mRNAs in the hippocampus of Aβ_1-42_-induced AD model rats, which is a useful experimental animal model of AD which emphasizes the inflammatory component of the disease pathology, and strongly complements the use of transgenic animal models in advancing our understanding of AD [16]. Next we performed Gene ontology (GO) and Kyoto Encyclopedia of Genes and Genomes (KEGG) analyses. We also constructed a circRNA-associated-ceRNA network. Findings arising from this study will expand our understanding of the potential role of the circRNA-associated-ceRNA network involved in AD pathogenesis and therapeutic strategy.

## RESULTS

### Altered circRNA, miRNA and mRNA profiles in the AD rat hippocampus

**Figure 1:**
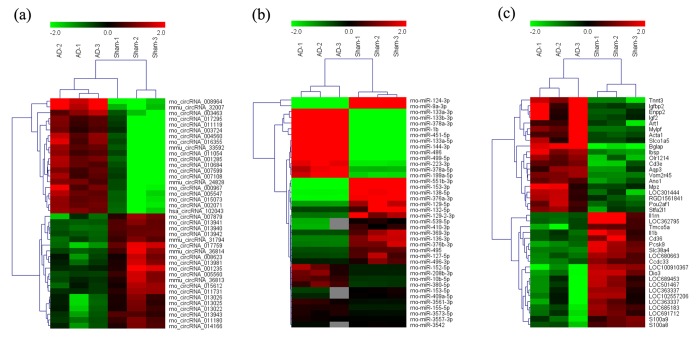
**Heat map of the top 40 differentially-expressed circRNAs** (**A**), miRNAs (**B**), and mRNAs (**C**) in AD hippocampal tissue. The data are depicted as matrices in which each row represents one circRNA, miRNA, or mRNA and each column represents one of the hippocampal samples. Relative circRNA, miRNA, or mRNA expression is depicted according to the color scale shown at the top. Red and green represent high and low relative expression, respectively; -2.0, 0, and 2.0 are fold-changes in the corresponding spectrum. The magnitude of deviation from the median is represented by color saturation.

**Figure 2:**
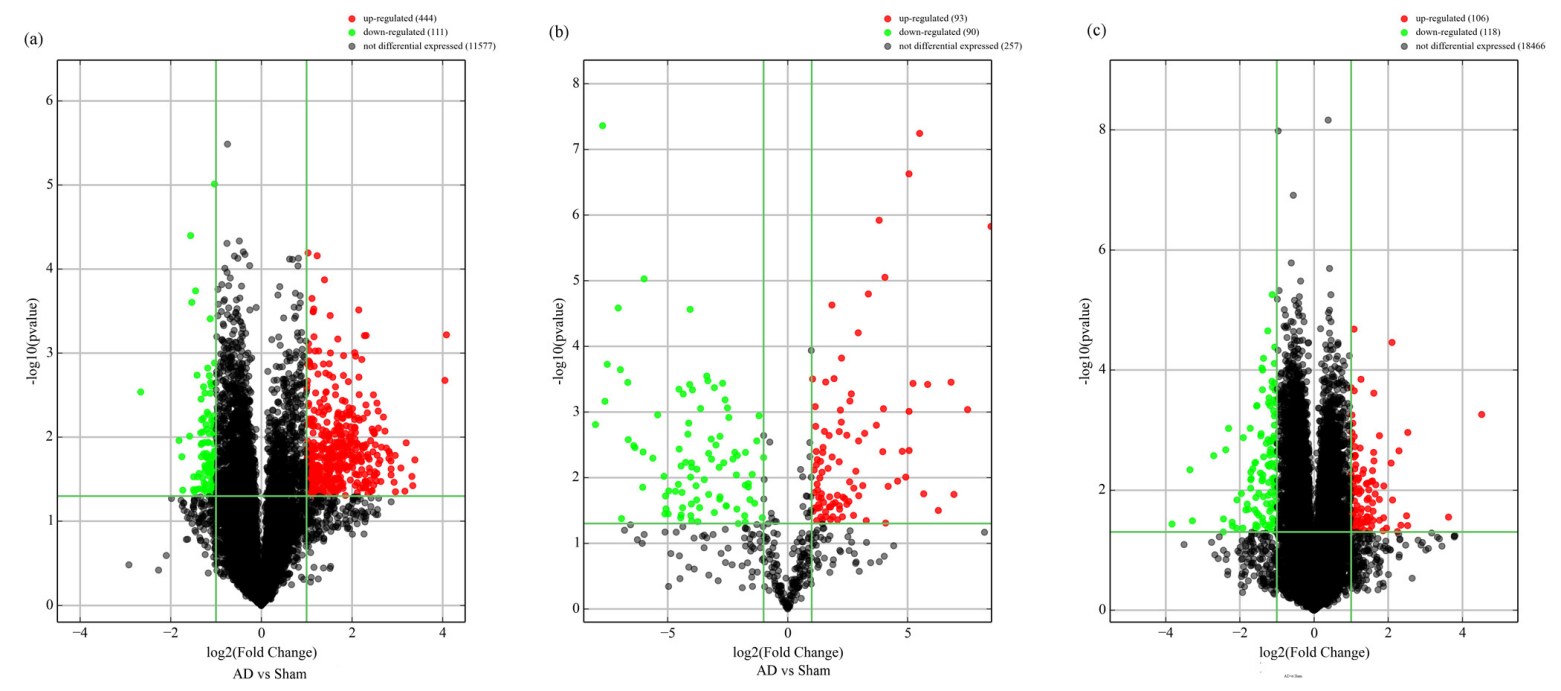
**Volcano plot of differentially-expressed circRNAs** (**A**), miRNAs (**B**), and mRNAs (**C**) between AD and sham hippocampal samples. Volcano plots were constructed using fold-change values and *p*-values. The vertical lines correspond to 2.0-fold up- and down-regulation between normal and AD samples (N *vs.* **D**), and the horizontal lines represent *p*-values. Red plot points represent differentially-expressed circRNAs with statistical significance.

The expression of circRNAs, miRNAs, and mRNAs in the hippocampus of AD rats were profiled using microarrays. Using a threshold of fold change (FC) ≥ 2.0 and a *p*-value < 0.05, 555 circRNAs, 183 miRNAs, and 319 mRNAs were significantly altered in the AD rat hippocampus compared to sham controls. The results showed that 444 circRNAs, 93 miRNAs, and 173 mRNAs were up-regulated, while 111 circRNAs, 90 miRNAs, and 146 mRNAs were down-regulated. The most up-regulated were circRNA_008964, miR-133a-5p, and BGLAP with FCs of 16.97, 355.67, and 22.96, respectively, whereas circRNA_017759, miR-551b-3p, and IL-1RN were the most down-regulated, with corresponding FCs of 6.32, 257.63 and 14.21. The top 20 up-regulated and 20 down-regulated circRNAs, miRNAs, and mRNAs in the AD group are listed in Tables 1-3. Hierarchical clustering and volcano plot visualization (Fig. 1 and 2) suggested that the expression level of circRNAs, miRNAs and mRNAs were distinguishable and variable.

**Table 1:** Top 40 differently expressed circRNAs in microarray analysis.

circRNA	p-value	FDR	Fold change	Regulation	chrom	strand	circRNA_type	Gene symbol
rno_circRNA_008964	0.000606	0.094365	16.9714	up	chr2	+	exonic	Ppp3ca
mmu_circRNA_32007	0.002111	0.108069	16.57521	up	chr18	-	sense overlapping	RGD1308601
rno_circRNA_004560	0.018609	0.182842	10.47134	up	chr14	-	exonic	RGD1305110
rno_circRNA_017295	0.037675	0.225271	10.14399	up	chr9	-	exonic	Clk1
rno_circRNA_016355	0.029281	0.207134	10.00513	up	chr8	-	sense overlapping	Birc2
rno_circRNA_001285	0.01166	0.16506	9.170892	up	chr1	+	exonic	Pdcd11
rno_circRNA_011119	0.043819	0.236427	8.989032	up	chr3	-	exonic	Ext2
mmu_circRNA_33592	0.023237	0.196417	8.459	up	chr3	-	exonic	Acvr1
rno_circRNA_003724	0.044537	0.237503	7.782604	up	chr12	-	exonic	RGD1309762
rno_circRNA_010684	0.016463	0.17835	7.75411	up	chr3	-	exonic	Golga1
rno_circRNA_011054	0.034331	0.216266	7.619158	up	chr3	+	intergenic	
rno_circRNA_007599	0.023829	0.197354	7.284635	up	chr18	+	sense overlapping	Zfp516
rno_circRNA_007108	0.028113	0.205101	7.274158	up	chr18	+	exonic	Wdr33
mmu_circRNA_24828	0.019181	0.184686	7.260869	up	chr6	+	sense overlapping	Tssc1
rno_circRNA_005547	0.015465	0.175514	7.112599	up	chr15	+	exonic	Mipep
rno_circRNA_000967	0.014718	0.173347	7.044118	up	chr1	+	exonic	Cpsf7
rno_circRNA_015073	0.017918	0.182842	7.039097	up	chr7	-	exonic	Polr3b
rno_circRNA_003463	0.013453	0.170196	6.770216	up	chr12	+	exonic	Slc46a3
rno_circRNA_002071	0.008468	0.149026	6.626886	up	chr10	+	exonic	Tekt5
hsa_circRNA_102043	0.00576	0.138131	6.473515	up	chr10	+	exonic	Acaca
rno_circRNA_017759	0.002899	0.111828	6.320172	down	chrX	+	intergenic	
mmu_circRNA_36814	0.01092	0.161814	3.519062	down	chr5	+	exonic	Rad23b
rno_circRNA_007879	0.017054	0.179628	3.370089	down	chr19	-	exonic	Nr3c2
rno_circRNA_001235	0.042661	0.234935	3.322749	down	chr1	-	exonic	Got1
rno_circRNA_005560	0.00972	0.154309	2.990798	down	chr15	+	exonic	Dpysl2
rno_circRNA_013940	0.00004	0.066585	2.94782	down	chr6	+	exonic	Ptprn2
rno_circRNA_013941	0.000249	0.077601	2.893453	down	chr6	+	exonic	Ptprn2
rno_circRNA_008623	0.042057	0.233777	2.8228	down	chr2	+	sense overlapping	Ash1l
rno_circRNA_013981	0.029554	0.207381	2.812952	down	chr6	-	exonic	Nrxn1
rno_circRNA_013026	0.044491	0.23747	2.785261	down	chr5	+	exonic	Rere
rno_circRNA_013942	0.000182	0.077601	2.733282	down	chr6	+	exonic	Ptprn2
rno_circRNA_013025	0.041907	0.233777	2.704128	down	chr5	+	sense overlapping	Rere
mmu_circRNA_31794	0.001829	0.105466	2.669903	down	chr18	+	exonic	Camk2a
rno_circRNA_015612	0.029545	0.207381	2.653884	down	chr7	-	exonic	Trps1
mmu_circRNA_36813	0.026985	0.203613	2.590337	down	chr5	+	exonic	Rad23b
rno_circRNA_013022	0.041158	0.23268	2.585987	down	chr5	+	exonic	Rere
rno_circRNA_013943	0.008876	0.15	2.525526	down	chr6	+	exonic	Ptprn2
rno_circRNA_011180	0.003519	0.119602	2.509546	down	chr4	-	exonic	Magi2
rno_circRNA_011731	0.012178	0.167316	2.50498	down	chr4	-	exonic	Grin2b
rno_circRNA_014166	0.017049	0.179628	2.501832	down	chr6	+	exonic	Dtnb

**Table 2:** Top 40 differently expressed miRNAs in microarray analysis.

Name	FC	P-value	FDR	Regulation	Sham -1	Sham -2	Sham-3	AD-1	AD-2	AD-3
rno-miR-133a-5p	355.6778	1.49E-06	0.000597	up	0.030016	0.024263	0.025	9.676819	9.532174	8.988871
rno-miR-133a-3p	180.9255	0.000918	0.023498	up	0.139021	0.29636	0.278125	52.46193	40.3513	36.27822
rno-miR-1b	121.9097	0.017959	0.081167	up	0.35545	0.110919	0.207813	37.47377	30.81565	13.89984
rno-miR-133b-3p	112.2112	0.000352	0.016336	up	0.255924	0.589255	0.5125	59.63959	45.44174	47.2655
rno-miR-152-5p	77.69073	0.031524	0.108617	up	0.006319	0.010399	0.025	1.654822	1.069565	0.516693
rno-miR-378a-5p	57.07956	0.00038	0.01635	up	0.053712	0.084922	0.040625	2.900169	3.384348	3.947536
rno-miR-208b-3p	50.91059	0.017594	0.080291	up	0.020537	0.025997	0.014063	1.159052	1.396522	0.529412
rno-miR-144-3p	45.24951	5.72E-08	6.88E-05	up	0.104265	0.220104	0.2125	8.164129	8.168696	7.960254
rno-miR-486	37.36758	0.000367	0.016336	up	0.1406	0.216638	0.253125	7.678511	8.709565	6.419714
rno-miR-499-5p	33.39683	0.003852	0.038921	up	0.349131	0.221837	0.19375	9.771574	9.996522	5.771065
rno-miR-10b-5p	33.32301	0.000977	0.023498	up	0.031596	0.025997	0.035938	1.219966	1.074783	0.82194
rno-miR-155-5p	33.28875	2.36E-07	0.000189	up	0.017378	0.015598	0.010938	0.49577	0.474783	0.491256
rno-miR-3561-3p	30.31098	0.009789	0.060993	up	0.022117	0.008666	0.014063	0.566836	0.526957	0.265501
rno-miR-3573-5p	27.40815	0.003971	0.039624	up	0.020537	0.003466	0.010938	0.358714	0.217391	0.381558
rno-miR-378a-3p	23.9381	0.011289	0.064185	up	0.93207	2.391681	1.076563	27.22673	27.89217	50.21622
rno-miR-223-3p	18.23247	0.013543	0.071138	up	0.265403	0.253033	0.314063	3.908629	7.328696	3.941176
rno-miR-3557-3p	16.94205	0.049043	0.137085	up	0.014218	0.003466	0.00625	0.218274	0.125217	0.062003
rno-miR-199a-5p	16.61341	8.89E-06	0.002256	up	0.306477	0.176776	0.207813	3.588832	3.928696	3.963434
rno-miR-451-5p	15.92029	0.00089	0.023498	up	1.391785	2.157712	1.814063	33.7868	28.18609	23.41653
rno-miR-380-5p	15.60035	0.004015	0.039631	up	0.047393	0.069324	0.101563	0.891709	1.481739	1.031797
rno-miR-551b-3p	0.003881	0.001551	0.027567	down	5.919431	8.823224	9.196875	0.033841	0.04	0.019078
rno-miR-153-3p	0.004766	4.37E-08	6.88E-05	down	7.663507	7.908146	7.759375	0.025381	0.036522	0.049285
rno-miR-539-5p	0.005103	0.000688	0.020933	down	1.507109	1.649913	1.375	0.00846	0.006957	N/A
rno-miR-376a-3p	0.005457	0.000188	0.012127	down	3.92891	5.07279	4.80625	0.018613	0.031304	0.025437
rno-miR-124-3p	0.007493	2.61E-05	0.003452	down	92.09321	80.76256	80.40625	0.592217	0.558261	0.747218
rno-miR-136-3p	0.007982	0.000226	0.013604	down	2.020537	2.564991	2.603125	0.025381	0.02087	0.011129
rno-miR-129-5p	0.008243	0.041911	0.124593	down	2.519747	6.67591	2.790625	0.047377	0.043478	0.007949
rno-miR-9a-3p	0.009872	0.000354	0.016336	down	41.50079	56.26516	48.03438	0.448393	0.542609	0.448331
rno-miR-410-3p	0.010002	0.002631	0.032583	down	1.919431	1.443674	1.782813	0.01692	0.017391	N/A
rno-miR-153-5p	0.011705	0.003247	0.035819	down	0.507109	0.582322	0.6875	0.001692	0.012174	N/A
rno-miR-409a-5p	0.012151	0.00348	0.036711	down	0.36019	0.298094	0.409375	0.001692	0.006957	N/A
rno-miR-138-5p	0.015186	0.014003	0.072249	down	8.292259	3.935875	4.757813	0.081218	0.095652	0.081081
rno-miR-369-3p	0.015371	0.004061	0.039771	down	1.259084	1.856153	2.292188	0.025381	0.024348	0.033386
rno-miR-495	0.015832	9.38E-06	0.002256	down	1.265403	1.377816	1.423438	0.023689	0.029565	0.011129
rno-miR-129-2-3p	0.020383	0.005048	0.043957	down	3.361769	1.859619	2.346875	0.052453	0.074783	0.027027
rno-miR-376b-3p	0.023408	0.001105	0.025237	down	1.962085	1.953206	2.729688	0.079526	0.052174	0.023847
rno-miR-127-5p	0.028105	0.00953	0.06067	down	1.232227	2.107452	1.13125	0.060914	0.050435	0.014308
rno-miR-132-5p	0.02883	0.035542	0.115982	down	1.14218	4.088388	3.13125	0.089679	0.078261	0.073132
rno-miR-496-3p	0.029383	0.019014	0.082393	down	0.50237	1.315425	0.948438	0.025381	0.04	0.015898
rno-miR-3542	0.029791	0.029799	0.10517	down	0.075829	0.152513	0.117188	0.003384	0.003478	N/A

**Table 3:** Top 40 differently expressed mRNAs in microarray analysis.

Genbank Accession	Gene Symbol	P-value	FDR	Fold Change	Regulation	AD-1	AD-2	AD-3	Sham -1	Sham -2	Sham -3
NM_013414	Bglap	0.00055	0.026406	22.96703	up	8.052033	9.051468	8.575123	4.18026	4.55122	3.382667
NM_001270665	Tnnt3	0.028149	0.128763	12.35968	up	5.806189	5.275991	8.642943	3.211319	3.209004	2.422092
NM_012587	Ibsp	0.001096	0.031424	5.771831	up	6.252816	5.279157	5.647549	3.384729	3.16391	3.043795
NM_013122	Igfbp2	0.038993	0.15308	5.754793	up	11.06608	9.303097	12.16206	8.300828	8.427517	8.228595
NM_012605	Mylpf	0.026781	0.125297	5.641142	up	7.466302	7.129157	9.462192	5.464603	5.479666	5.625421
NM_057104	Enpp2	0.038489	0.15198	5.07149	up	12.46112	10.68197	13.24472	9.646646	9.645828	10.0681
NM_001000438	Olr1214	0.002217	0.039344	4.895564	up	5.185619	4.642926	5.070082	2.360833	3.241277	2.422092
NM_031511	Igf2	0.049039	0.175423	4.757985	up	13.65679	11.66636	14.31541	10.78843	10.91098	11.1881
XR_086340	LOC301444	0.014615	0.090422	4.340022	up	10.78893	10.89127	9.746195	8.448961	8.932608	7.691718
XM_001059752	Vom2r45	3.47E-05	0.016	4.296108	up	4.366794	4.380062	4.672477	2.360833	2.327317	2.422092
NM_001108140	Cd3e	0.003543	0.047336	4.22607	up	4.738903	4.657002	4.962316	2.360833	2.402169	3.357269
NM_001107541	Art1	0.043071	0.162562	3.875367	up	4.757209	4.724255	6.257683	3.799408	3.654648	2.422092
NM_031703	Aqp3	0.008524	0.068504	3.685033	up	3.96804	4.769804	4.846925	2.360833	2.390517	3.188386
BC078859	Mpz	0.028946	0.130331	3.67334	up	7.999598	7.897556	6.286076	5.421919	5.426666	5.703367
NM_019212	Acta1	0.047568	0.17213	3.632821	up	6.637662	6.667013	8.622218	5.496824	5.344661	5.502137
NM_030838	Slco1a5	0.031783	0.137186	3.400608	up	8.433027	7.296741	9.147305	6.444243	6.437362	6.69809
NM_001107564	Ano1	0.001239	0.03245	3.397198	up	8.816945	9.067116	8.695915	6.800028	7.044566	7.442346
XM_227107	RGD1561841	0.014316	0.089396	3.391897	up	10.07803	10.32065	9.068267	8.393194	8.027146	7.760331
NM_001109599	Pou2af1	0.008157	0.067119	3.171449	up	3.934713	4.788568	3.704984	2.360833	2.649913	2.422092
NM_001004129	Stfa2l1	0.011619	0.07994	3.137655	up	7.900152	8.128724	7.293016	5.570128	6.459183	6.343522
NM_022194	Il1rn	0.036753	0.148451	14.21197	down	5.016814	4.992311	5.159386	9.625172	10.56531	6.465138
XR_361775	LOC100910367	0.004583	0.052819	10.16713	down	3.468741	4.307965	2.321873	6.875965	6.716166	6.54397
BC098733	LOC362795	0.032448	0.138888	9.708935	down	6.844371	6.675695	7.021456	10.91333	11.35412	8.112008
NM_017210	Dio3	0.002669	0.042782	6.519498	down	6.231844	6.621063	5.503228	9.068267	9.062721	8.339429
XM_006255043	LOC689453	0.049874	0.177224	5.455306	down	5.015599	5.841514	3.128502	7.675495	7.21799	6.435111
XM_006227100	LOC501467	0.030232	0.133751	5.431563	down	5.4069	6.083151	3.794581	8.035631	7.5748	6.998302
NM_199253	Pcsk9	0.002133	0.039056	5.204765	down	2.426524	2.665853	2.946293	5.604621	5.012788	4.56076
NM_130748	Slc38a4	0.000938	0.030292	4.934402	down	4.587482	4.637961	4.475671	7.354593	6.783875	6.471272
NM_031512	Il1b	0.034329	0.143302	4.617557	down	4.037401	4.364745	3.874776	7.502599	6.263231	5.132481
NM_001014221	LOC363337	0.039423	0.154047	4.587823	down	8.473712	9.35102	7.109605	10.83744	10.83451	9.855817
XR_349427	LOC102557206	0.043126	0.162711	4.261659	down	7.166277	7.946725	5.904576	9.512047	9.477423	8.302353
XM_006224554	Tmco5a	0.014583	0.090373	4.222635	down	5.590317	4.665117	6.381915	7.747607	7.674106	7.450068
XM_006255374	LOC685183	0.011431	0.079358	3.885825	down	4.719262	5.091599	3.778711	6.874688	6.423315	6.166232
NM_001014221	LOC363337	0.042926	0.162322	3.775706	down	9.380106	10.53603	8.459474	11.81476	11.39776	10.91333
NM_001127377	LOC680663	0.001338	0.033124	3.748348	down	3.41188	3.828432	3.532797	5.766442	5.629198	5.096233
NM_031561	Cd36	0.021348	0.110965	3.577859	down	4.227085	4.462658	4.176351	5.902704	7.071649	5.40903
XM_006222689	LOC691712	0.017104	0.098359	3.482986	down	4.723632	5.200954	3.846046	6.733775	6.481439	5.956392
NM_053587	S100a9	0.02089	0.109858	3.409311	down	5.465103	4.943009	3.967565	6.199166	6.637269	6.847683
NM_001014091	Ccdc33	0.000936	0.030292	3.293413	down	2.477567	2.602366	2.926151	4.675579	4.253647	4.235609
NM_053822	S100a8	0.006707	0.062417	3.266306	down	5.768222	5.836943	4.862041	7.00802	7.213229	7.368936

### Expression profile validation

**Figure 3:**
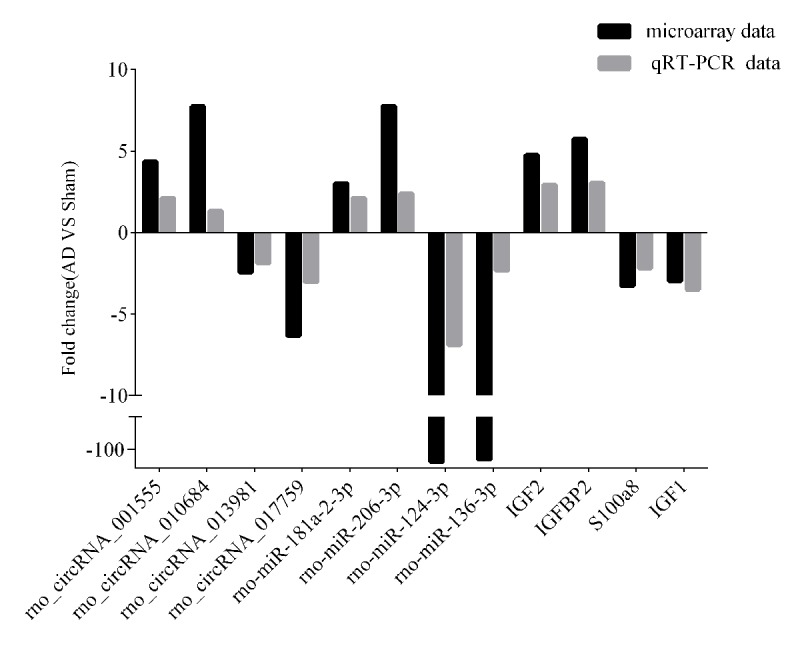
**qRT-PCR-validated ncRNA and mRNA expression changes.** Microarray validation by qRT-PCR. Expression levels of four circRNAs, four miRNAs, and four mRNAs were determined by qRT-PCR. Each assay was performed at least three times. *p < 0.05.

To validate the accuracy and reliability of the microarray profiling data, some transcripts, including four circRNAs (rno_circRNA_001555, rno_circRNA_010684, rno_circRNA_01398, and rno_circRNA_017759), four miRNAs (rno-miR-181a-2-3p, rno-miR-124-3p, rno-miR-136-3p, and rno-miR-206-3p), and four mRNAs (IGF2, IGFBP2, S100a8, and IGF1) were randomly selected for quantitative real-time polymerase chain reaction (qRT-PCR) analysis in nine samples including those used for microarray analysis. As shown in Fig. 3, the microarray data were consistent with the qRT-PCR results in terms of the expression levels of the validated ncRNAs and mRNAs.

### GO and KEGG pathway analyses of differentially-expressed mRNAs

**Figure 4:**
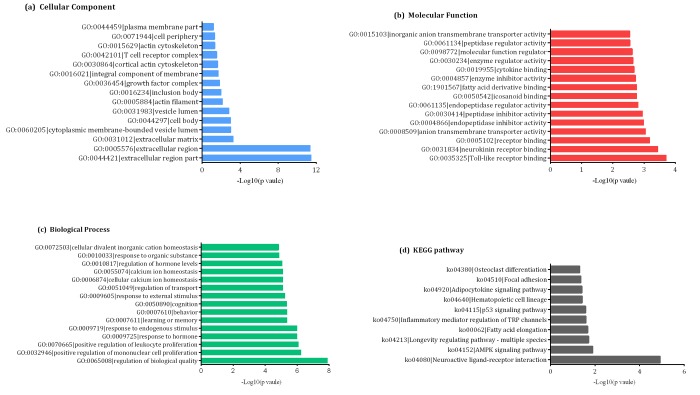
**Gene ontology (GO) enrichment and pathway analysis of differentially- expressed mRNAs** showing the most significantly enriched (-log10, *p*-value) GO terms of differentially-expressed mRNAs according to (**A**) cellular components, (**B**) molecular functions, and (**C**) biological processes. (**D**) The top ten enrichment scores (-log10, *p*-value) of significantly enriched KEGG pathways.

GO and KEGG pathway analyses allow for the functional annotation of differentially-expressed mRNAs. GO analysis indicated that the most enriched mRNAs correlated with the extracellular region part (GO: 0044421) of the cellular component analysis (Figure 4a). Meanwhile, the majority of genes were related to toll-like receptor binding (GO:0035325) in the molecular functions and regulation of biological quality (GO:0065008) aspect of the biological processes analyses (Figure 4b and 4c). KEGG pathway analysis indicated that 10 KEGG pathways (*p* < 0.05) were associated with dysregulated mRNAs involved in neuroactive ligand-receptor interaction, AMP-activated protein kinase (AMPK) signaling pathway, longevity regulating pathway-multiple species, fatty acid elongation, inflammatory mediator regulation of transient receptor potential (TRP) channels, p53 signaling pathway, hematopoietic cell lineage, adipocytokine signaling pathway, focal adhesion and osteoclast differentiation (Figure 4d).

### Prediction of circRNA-miRNA interactions

**Figure 5:**
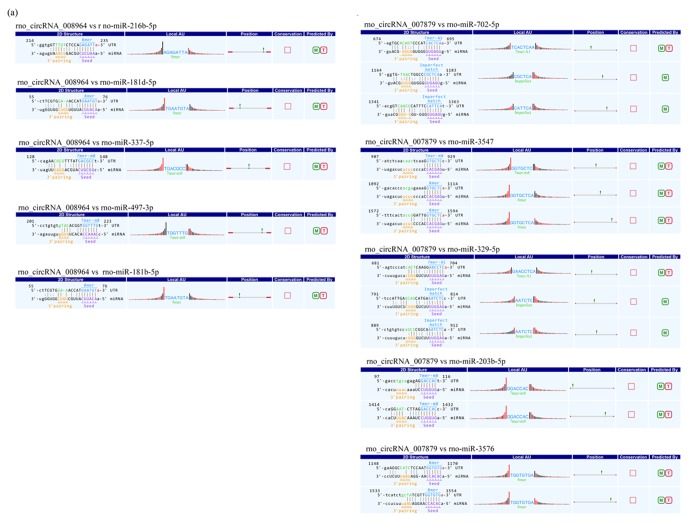
**Example of detailed annotation for circRNA-miRNA interactions.** (**A**) rno_circRNA_008964. (**B**) rno_circRNA_017759. 8mer: bases from number 2 to 8 matched perfectly, and number 1 base is A; 7mer-m8: bases from number 2 to 8 matched perfectly, and number 1 base is not A; 6mer: bases from number 2 to 7 matched perfectly, and number 1 base is not A; offset 6mer: bases from number 3 to 8 matched perfectly; imperfect match: there is imperfect base match from number 2 to 7; M: circRNA-miRNA interaction can be predicted by miRanda; T: circRNA-miRNA interaction can be predicted by TargetScan.

To determine the function of circRNA, interactions between circRNAs and their target miRNAs were theoretically predicted by conserved seed-matching sequences. To find the potential miRNA target, two confirmed circRNAs (rno_circRNA_008964 and rno_circRNA_017759) were selected, and circRNA-miRNA interaction was predicted using Arraystar's miRNA target prediction software based on the TargetScan and miRanda databases. The potential miRNA targets of rno_circRNA_008964 included rno-miR-216b-5p, rno-miR-181d-5p, rno-miR-337-5p, rno-miR-497-3p, and rno-miR-181b-5p (Fig. 5a). For rno_circRNA_007879, the potential miRNA targets included rno-miR-702-5p, rno-miR-3547, rno-miR-329-5p, rno-miR-203b-5p and rno-miR-3576 (Fig. 5).

### Construction of a circRNA-miRNA regulatory network

**Figure 6:**
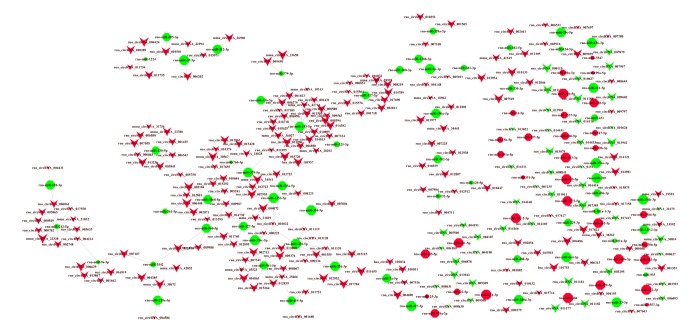
**CircRNA-miRNA network analysis.** A total of 245 circRNAs and 144 miRNAs containing 279 relationships were selected to generate a network map. The circRNA-miRNA co-expression network was constructed with Cytoscape V3.5.0 software. Within this network, V-shaped nodes represent circRNA and octagon nodes represent miRNA. Red and green represent up- and down-regulation, respectively. Node size represents *p*-values (larger nodes for more significant *p*-values).

A circRNA-miRNA regulatory network, based on the microarray results, was constructed containing 245 circRNAs, 144 miRNAs and 279 relationships (Fig. 6). We observed that one circRNA could regulate multiple miRNAs in different ways, while one miRNA could be regulated by multiple circRNAs. For example, circRNA_34441 was co-related to four dysregulated miRNAs and MiR-153-5p was co-related with 25 dysregulated circRNAs. Thus, there appears to be a complex circRNA-miRNA regulatory network involved in the pathogenesis of AD.

### Construction of a circRNA-miRNA-mRNA regulatory network

**Figure 7:**
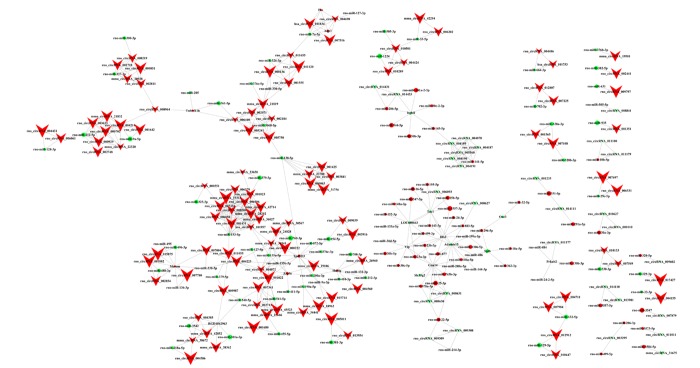
**CircRNA-miRNA-mRNA regulatory network.** The regulatory network consists of 140 circRNAs, 140 miRNAs, and 20 genes with 503 relationships. V-shaped, octagon, and diamond nodes represent circRNA, miRNA, and mRNA, respectively. Red and green represent up- and down-regulation, respectively. Node size represents *p*-values (larger nodes for more significant *p*-values).

To explore the molecular mechanism of ncRNAs, a circRNA-miRNA-mRNA regulatory network was constructed with 140 circRNAs as decoys, 140 miRNAs as centres, and 20 mRNAs as targets based on the microarray data (Fig. 7). Because there are binding sites between circRNAs and miRNAs, circRNAs could indirectly regulate miRNA target genes by competitively binding to miRNA as a miRNA sponge. For instance, we focused on miR-7a-5p, circRNA_101834 and circRNA_004690 that could regulate AQP3 expression by competing miRNA response elements (MREs) of miR-7a-5p. These data suggested that circRNAs harbor MREs and play pivotal regulatory roles in AD.

## DISCUSSION

To the best of our knowledge, this is the first integrated microarray analysis of circRNA, miRNA and mRNA expression profiles in the hippocampus of Aβ_1-42_-induced AD model rats. With FC ≥ 2.0 and *p*-value < 0.05 thresholds, 444 up-regulated and 111 down-regulated circRNAs, 93 up-regulated and 90 down-regulated miRNAs, and 173 up-regulated and 146 down-regulated mRNAs showed significant differential expression between the AD and control groups. These transcripts are thought to be associated with the pathogenesis of AD. For instance, the S100A8 precedes Aβ plaque formation [17], IGFBP-2 drives AD neurodegeneration [18], miR-146a-5p facilitates neuroinflammation in AD pathogenesis [19], and miR-132-3p contributes to tau hyper-phosphorylation [20]. Our qRT-PCR validation showd that the qRT-PCR results and microarray data were consistent, indicating that the latter were reliable.

To better understand the biological functions and potential mechanisms of mRNAs in the pathogenesis of AD, we performed GO and KEGG pathway analysis. Among the GO terms found in this study, the extracellular region part (GO:0044421), toll-like receptor binding (GO:0035325), and the regulation of biological quality (GO:0065008) have been reported to play important roles in AD development. Remarkable among the KEGG pathways were the AMPK and p53 signaling pathways, both known to potentially mediate AD pathogenesis [21–23].

Increasing lines of evidence indicate that circRNAs can function as endogenous sponges to influence miRNA activity, thereby regulating other transcripts containing miRNA-binding sites [24,25]. Therefore, we examined circRNA-miRNA interactions and found that the majority of circRNAs contained one or more miRNA binding sites based on sequence analyses. The association of miRNAs with AD indicated that circRNAs might have a regulatory role in AD. For example, rno_circRNA_003295 is potentially able to interact with rno-miR-206-3p, rno_circRNA_002441 with miR-144-3p, and rno_circRNA_012846 with rno-miR-10a-5p. Moreover, we constructed a circRNA-miRNA regulatory network to investigate potential relationships among the circRNAs and miRNAs. Our results indicated that circRNA-miRNA regulatory networks might play important roles in the pathogenesis of AD. However, beyond acting as a miRNA sponge, circRNAs might also influence mRNA transcriptional levels by interacting with the Pol II complex in the nucleus [26], or by directly binding to RNA-binding proteins and RNA-associated proteins to form RNA-protein complexes [27]. Furthermore, circRNAs can act on gene expression trans-functionally by competing with pre-mRNA splicing machinery [28], and endogenous circRNAs can be used as templates to encode proteins [29]. Therefore, the biological function of circRNA in the pathogenesis of AD requires further investigation.

Perturbations in competing endogenous RNA (ceRNA) regulatory networks (ceRNETs) including mRNAs, miRNAs, and circRNAs have been proposed to play critical roles in the pathogenesis of human disease [30–32]. For example, the circRNA-7/miRNA-7/UBE2A signaling pathway is significantly dysregulated in AD [33,34]. The application of ceRNET analysis may provide a novel view of interplay between ncRNAs and mRNAs, thereby offering some insight into molecular pathways. Our results provide the first assessment of ceRNETs in AD and suggest that specific ceRNETs are involved in disease etiology and pathogenesis.

There are certain limitations to our study which should be considered when interpreting our findings. Firstly, the sample size was limited, which could have resulted in under- or over-estimation of the numbers of altered circRNAs, miRNAs, and mRNAs. Larger sample sizes are needed to confirm our findings. Secondly, our results refer only to hippocampal samples from Aβ_1-42_-induced AD rats. Further research with other AD models, using additional sample sources such as blood and cerebrospinal fluid, is now needed to more accurately capture the pathophysiology of AD. Thirdly, our analyses were performed using gene expression microarrays with limited dynamic range that lack the ability to identify novel features. RNA-sequence technology should be utilized to unravel previously inaccessible transcriptome complexities. Finally, because the functions of circRNAs and miRNAs remain largely unknown, the interpretation of our data was not straightforward. Thus, we only predicted the functions of differentially-expressed mRNAs. Future studies that overcome such limitations are now highly warranted.

In conclusion, we identified dysregulated expression profiles of circRNAs, miRNA, and mRNAs in the hippocampus of AD rats, and present an innovative data integration analysis of circRNAs, miRNAs and mRNAs. Our results indicate that ncRNAs may interact to regulate the expression of their target protein-genes involved in the pathogenesis of AD. The next step is to validate and expand these findings in future studies, which might ultimately enable us to fully elucidate the mechanisms underlying AD.

## MATERIALS AND METHODS

### Ethics statement

All animal protocols were approved by the Central South University (Changsha, China) Institutional Review Board and were performed in compliance with the National Institutes of Health Guide for the Care and Use of Laboratory Animals. This investigation was conducted in accordance with appropriate ethical standards and the Declaration of Helsinki, as well as national and international guidelines.

### Brain samples

All hippocampal tissue samples were from brain tissues obtained in our previous study [6]. Briefly, 20 adult male Sprague Dawley rats (250 ± 30 g) were randomly divided into AD (n = 10) and sham (n = 10) groups. We performed intracerebroventricular injections of Aβ_1-42_ oligomers into the cerebral ventricles to induce a validated AD model, as previously described [35,36]. Briefly, the animals were anaesthetized and placed in a stereotactic frame, then the Aβ1–42 oligomers were injected bilaterally into the lateral ventricles through a stainless-steel cannula.

### RNA extraction

Total RNA was extracted from each hippocampal tissue sample by soaking in TRIzol Reagent (Invitrogen, Carlsbad, CA, USA) in accordance with the manufacturer’s instructions, which mainly includes homogenization, phase separation, RNA precipitation, RNA wash and RNA solubilization. RNA quantity and quality were measured using a NanoDrop ND-1000 (Thermo Scientific, Waltham, MA, USA), and RNA integrity was assessed by standard denaturing agarose gel electrophoresis.

### Microarray analysis

Sample labelling and microarray hybridization for gene expression were performed according to the Agilent One-Color Microarray-Based Gene Expression Analysis protocol (Agilent Technology, Santa Clara, CA, USA), which included RNA purification, transcription into fluorescent-labeled cRNA, and hybridization onto the Rat 4x44K Gene Expression Array (Agilent). Finally, the hybridized arrays were washed, fixed, and scanned using the Agilent DNA Microarray Scanner G2505C. RNA labeling and array hybridization for miRNA analysis were conducted following the Exiqon manual (Vedbaek, Denmark). After quality control, the miRCURY™ Hy3™/Hy5™ Power labeling kit (Exiqon) was used for miRNA labeling according to the manufacturer guidelines. After stopping the labeling procedure, Hy3™-labeled samples were hybridized on the Rat miRCURY LNA™ microRNA Array 7th Gen (Exiqon), according to the array manual. The slides were scanned using the Axon GenePix 4000B microarray scanner (Axon Instruments, Foster City, CA, USA). circRNA sample preparation and microarray hybridization were performed based on Arraystar standard protocols (Super RNA Labeling Kit; Arraystar, Rockville, MD, USA). Briefly, total RNAs were digested with Rnase R (Epicentre, Inc., Madison, WI, USA) to remove linear RNAs and enrich for circular RNAs. Then, the enriched circular RNAs were amplified and transcribed into fluorescent-labeled cRNA using a random priming method (Super RNA Labeling Kit). The labeled cRNAs were hybridized onto the Arraystar Rat circRNA Array (8x15K, Arraystar). After washing the slides, the arrays were scanned using the Agilent Scanner G2505C. All data collection was performed using Agilent Feature Extraction software (version 11.0.1.1). KangChen Bio-tech (Shanghai, China) performed all microarray analyses.

### Quantitative real-time polymerase chain reaction (qRT-PCR) validation

As previously described [31], total RNA was isolated using TRIzol Reagent, and then reverse-transcribed into cDNA using SuperScript III Reverse Transcriptase (Invitrogen) according to the manufacturer’s instruction. An Applied Biosystems ViiA™7 Real-Time PCR System and 2× PCR Master Mix were used to perform qRT-PCR (Arraystar) in accordance with the manufacturer's instructions. The relative circRNA and mRNA expression levels were calculated using the 2^-ΔΔCt^ method and were normalized to GAPDH as an endogenous reference transcript [37]. miRNA expression levels were normalized to that of U6. The specific primers for each gene are listed in Table 4. Data shown represent the means of three experiments.

**Table 4:** Primers designed for qRT-PCR validation of candidate circRNAs, miRNAs, and mRNAs.

	Forward primer	Reverse Primer	PrProduct length (bp)	Tm(°C)
rno_circRNA_001555	5’- ATGAGCAATGACTCCCCAGAA-3’	5’- GAGAGTATGGTCTGTTGCGTTG-3’	60	60
rno_circRNA_010684	5’- TGGATCTAAAGCAGCTACAGAA-3’	5’- CTTTGGTTCCATTCATCCTTAT-3’	82	60
rno_circRNA_013981	5’- CTACCTTGAGCTGCACATACTG-3’	5’- TTTGTCCACCACCTTTGCT-3’	58	60
rno_circRNA_017759	5’- GAGTATCCACTGGTGACGACTG-3’	5’- AATATGCTGATCTTGTTTTCACC-3’	69	60
rno-miR-181a-2-3p	5’-GGACCACTGACCGTTGAC-3’	5’-CAGTGCGTGTCGTGGAG-3’	64	60
rno-miR-206-3p	5’- TGGGGTGGAATGTAAGGAAGT-3’	5’-CAGTGCGTGTCGTGGAGT-3’	65	60
rno-miR-124-3p	5’-GGGTAAGGCACGCGGT-3’	5’-GTGCGTGTCGTGGAGTCG-3’	61	60
rno-miR-136-3p	5’- GGGGACATCATCGTCTCAAAT -3’	5’-CAGTGCGTGTCGTGGAGT-3’	65	60
IGFBP2	5’- TCTACTCCCTGCATATCCCCA-3’	5’- GGTTCACACACCAGCACTCC-3’	105	60
IGF2	5’- GCTTGTTGACACGCTTCAGTT-3’	5’- TAGACACGTCCCTCTCGGA-3’	179	60
IGF1	5’- GGGCATTGTGGATGAGTGTTG -3’	5’- GCTGGGACTTCTGAGTCTTGG -3’	148	60
S100A8	5’-GGGAATCACCATGCCCTCTAC-3’	5’-GCCCACCCTTATCACCAACAC-3’	168	60

### GO annotations and KEGG pathway analyses

GO annotations and KEGG pathway analyses were performed to investigate the roles of all differentially-expressed mRNAs, as previously described [38,39]. Briefly, GO analysis was applied to elucidate genetic regulatory networks of interest by forming hierarchical categories according to the molecular functions, biological processes, and cellular component aspects of the differentially expressed genes (http://www.geneontology.org). The -log10 (*p*-value) denotes enrichment scores that represent the significance of GO term enrichment among differentially-expressed genes. KEGG pathway analyses were performed to explore significant pathways associated with the differentially-expressed genes (http://www.genome.jp/kegg/). The -log10 (*p*-value) denotes an enrichment score for the significance of pathway correlations.

### Annotation for circRNA-miRNA interaction

As described previously [40,41], circRNA-miRNA interactions were predicted with Arraystar's home-made miRNA target prediction software based on TargetScan (http://www.targetscan.org/) and miRanda (www.microrna.org/). The top five putative target miRNAs were identified. Then, we constructed a circRNA-miRNA regulatory network using the Cytoscape software V3.5.0 (San Diego, CA, USA).

### CircRNA-associated ceRNA network construction

The circRNA-associated ceRNA network was constructed and visually displayed using the Cytoscape software V3.5.0 (San Diego, CA, USA) based on microarray data analysis results, as previous described [42]. Different shapes and colors represent different RNA types and regulated relationships, respectively. Node size was inversely proportion to the *p*-value.

### Statistical analysis

All data were analysed using SPSS version 22.0 software (IBM Corp. Armonk, NY, USA) and presented as mean ± standard error of the mean (SEM). Student’s t-tests were used for comparisons between two groups, whereas one-way analysis of variance was performed for repeated measures. False discovery rates were calculated to correct *p-*values. Differences with *p* < 0.05 were considered to be statistically significant. Fold changes (FCs) and Student’s t-tests were used to determine the statistical significance of the microarray results. FC ≥ 2 and *p* < 0.05 were used as thresholds for designating differentially-expressed ncRNAs and mRNAs.
